# Decline in macrolide resistance rates among *Streptococcus pyogenes* causing pharyngitis in children isolated in Italy

**DOI:** 10.1007/s10096-015-2414-x

**Published:** 2015-05-30

**Authors:** G. Gherardi, D. Petrelli, M. C. Di Luca, F. Pimentel de Araujo, P. Bernaschi, A. Repetto, J. Bellesi, L. A. Vitali

**Affiliations:** University Campus Bio-Medico, 00128 Roma, Italy; School of Biosciences and Veterinary Medicine, University of Camerino, 62032 Camerino, MC Italy; Unit of Microbiology, Bambino Gesù Children’s Hospital, Roma, Italy; Azienda Ospedaliera di Perugia, Struttura Complessa di Microbiologia, Santa Maria della Misericordia Hospital Perugia, Perugia, Italy; Azienda Ospedaliera di Macerata, Macerata, Italy; School of Pharmacy, Microbiology Unit, University of Camerino, via Gentile III da Varano, 62032 Camerino, MC Italy; Department of Microbiology and Infection Control, University Hospital of North Norway, 9038 Tromsø, Norway; Department of Infectious, Parasitic and Immune-Mediated Diseases, Istituto Superiore di Sanità, Viale Regina Elena 299, 00161 Rome, Italy

## Abstract

Macrolides are often used to treat group A streptococcus (GAS) infections, but their resistance rates reached high proportions worldwide. The aim of the present study was to give an update on the characteristics and contemporary prevalence of macrolide-resistant pharyngeal GAS in Central Italy. A total of 592 isolates causing pharyngitis in children were collected in the period 2012–2013. Clonality was assessed by *emm* typing and pulsed-field gel electrophoresis (PFGE) for all macrolide-resistant strains and for selected susceptible isolates. Genetic determinants of resistance were screened by polymerase chain reaction (PCR). Forty-four GAS were erythromycin-resistant (7.4 %). Among them, 52.3 % and 50 % were clindamycin- and tetracycline-resistant, respectively. *erm*(B)-positive isolates (52.3 %) expressed the constitutive cMLS_B_ phenotype. *mef*(A) and its associated M phenotype were recorded in 40.9 % of the cases. The remaining *erm*(A)-positive isolates expressed the iMLS_B_ phenotype. Seventeen tetracycline-resistant isolates carried *tet*(M) and five isolates carried *tet*(O). Twenty-five *emm* types were found among all strains, with the predominance of *emm* types 12, 89, 1, and 4. Eleven *emm* types and 12 PFGE clusters characterized macrolide-resistant strains, with almost two-thirds belonging to *emm*12, *emm*4, and *emm*11. Macrolide-susceptible and -resistant *emm* types 12, 89, 11, and 4 shared related PFGE profiles. There was a dramatic decline in macrolide resistance in Central Italy among pharyngeal GAS isolates in 2012–2013 when compared to previous studies from the same region (*p* < 0.05), although macrolide consumption remained stable over the past 15 years. We observed a decrease in the proportion of macrolide-resistant strains within *emm* types commonly associated with macrolide resistance in the past, namely *emm*12, 1, and 89.

## Introduction

*Streptococcus pyogenes*, or Lancefield group A streptococcus (GAS), is an important pathogen implicated in a wide variety of human infections. The species is associated with both non-invasive diseases, such as acute pharyngitis, an infection for which it is the most common bacterial agent, and invasive infections, such as skin and soft-tissue infections, necrotizing fasciitis, bacteremia, sepsis, and toxic shock syndrome [1].

GAS remains sensitive to β-lactams, which is the drug class of choice in the treatment of most streptococcal infections because of its narrow spectrum of action and its efficacy in the prevention of post-streptococcal sequelae, such as rheumatic fever [1]. Macrolides have been recommended for patients allergic to β-lactams, and clindamycin is the preferred antibiotic in the treatment of patients with serious soft-tissue infections because of its ability to inhibit the production of several streptococcal virulence factors [1]. Resistance to erythromycin and related antibiotics has represented an important cause of concern [2, 3] and is mainly associated with two mechanisms. The first is expressed by *mef* genes, such as *mef*(A), encoding for an efflux pump, which confers resistance to 14- and 15-membered ring macrolides and susceptibility to clindamycin (M phenotype) [4]. The second mechanism involves *erm* genes, including *erm*(A) [subclass *erm*(TR)] and *erm*(B), which encode methylases targeting 23S rRNA [5]. The modification is associated with a decreased binding of all macrolides, lincosamides, and streptogramin type B to their targets on the ribosomal RNA (MLS_B_ phenotype), and it can be either induced (iMLS_B_ phenotype) or constitutive (cMLS_B_ phenotype). Other less common mechanisms of macrolide resistance are associated with mutations in the 23S rRNA gene sequence and/or alterations in riboproteins L4 and L22 [5]. Furthermore, an association between erythromycin resistance and cell invasiveness has been observed [6].

The major factor thought to be influencing the prevalence of macrolide resistance is macrolide consumption [7]. In addition, GAS clones showing *emm* types strongly associated with erythromycin resistance may contribute to the overall prevalence of macrolide resistance [8]. In this respect, Italy has always been highly ranked in the list of countries for macrolide resistance rates, ranging between 16 % and 36 % in Central Italy [9–11].

The aim of the present study was to examine the prevalence and phenotypic and genotypic characteristics of macrolide resistance in pharyngeal GAS isolates causing pharyngitis collected from children during two respiratory seasons (2012 and 2013) in Central Italy and compare our results with data published worldwide. Antimicrobial resistance phenotypes and genotypes were defined; the overall genotypes of strains were determined by *emm* typing and pulsed-field gel electrophoresis (PFGE).

## Materials and methods

### Bacterial isolates

GAS strains were collected from symptomatic individuals with pharyngotonsillitis from three different hospitals located in Central Italy during the respiratory season (January to June) in the years 2012 and 2013. One center is the largest referral pediatric hospital situated in Rome, while the others are general hospitals placed in Macerata and Perugia (Central Italy). All non-duplicate pharyngeal GAS clinical isolates included in the study were identified by colony morphology, β-hemolysis on blood agar, latex agglutination test (Streptococcal Grouping Kit, Oxoid, Basingstoke, UK), and susceptibility to bacitracin disks (10 U, Oxoid, Basingstoke, UK).

### Antimicrobial susceptibility testing

Antimicrobial susceptibility was determined by disk diffusion on Mueller–Hinton agar supplemented with 5 % defibrinated horse blood and 20 mg/L β-NAD, according to the guidelines and interpretative criteria of the European Committee on Antimicrobial Susceptibility Testing (EUCAST). The following antibiotic disks were included (Oxoid, Basingstoke, UK): penicillin G, erythromycin, clindamycin, tetracycline, norfloxacin, rifampicin, quinupristin/dalfopristin, and linezolid. Norfloxacin disks were used to screen for generic fluoroquinolone resistance. Isolates categorized as norfloxacin-susceptible were considered susceptible to all fluoroquinolones. The double-disk diffusion test was used to assign the strain to either the constitutive macrolide–lincosamide–streptogramin B (cMLS_B_), the inducible MLS_B_ (iMLS_B_), or the M phenotype [10].

### Detection of genetic determinants of antimicrobial resistance

Total bacterial DNA was extracted by the GenElute™ Bacterial Genomic DNA Kit (Sigma-Aldrich, St. Louis, MO, USA). Erythromycin-resistant isolates were studied for the presence of the macrolide resistance genes *erm*(A) [subclass *erm*(TR)], *erm*(B), and *mef*(A) by polymerase chain reaction (PCR) [12]. Erythromycin-resistant GAS being resistant also to tetracycline were analyzed by PCR to determine the presence of the resistance genes *tet*(M) and *tet*(O) [13].

### *emm* typing

Isolates were *emm* typed according to protocols and recommendations by the *Streptococcus* Laboratory at the Centers for Disease Control and Prevention (CDC, Atlanta, GA, USA). Assignment of the *emm* type is achieved by the comparison of the query sequence with the *emm* type reference database. The analysis considers 90 bases encoding the N-terminal part of the mature M protein.

### PFGE macrorestriction analysis

Total DNA from all erythromycin-resistant and a selected subgroup of erythromycin-susceptible isolates was extracted and digested with 20 U of *Sma*I (Fermentas, Vilnius, Lithuania), as previously described [12]. As for DNA of isolates not digested with *Sma*I, the restriction enzyme *Cfr9*I, an isoschizomer of *Sma*I, was used, as previously described [14]. DNA bands were resolved and interpreted according to previously reported criteria [15]. Briefly, isolates with identical profiles were assigned to the same PFGE type; isolates with profiles differing by 1 to 6 bands were assigned to different subtypes within the same PFGE type; isolates with profiles showing more than 6 bands of difference were considered unrelated.

### Statistical analysis

Statistical analysis was performed by Centurion XV software (STATGRAPHICS). Comparison of partitions and pairwise agreement measures were done according to Silva-Costa et al. [8] and expressed as adjusted Wallace coefficients.

## Results

### Antibiotic resistance rates, phenotypes, and genotypes

A total of 592 GAS isolates were collected from children with pharyngitis during the periods January–June 2012 and 2013. Patients were aged between 2 and 13 years (mean = 6.7 years, standard deviation =2.7 years), and 56 % were males. A total of 44 GAS were erythromycin-resistant, with an overall rate of erythromycin resistance of 7.4 %. Over the 2-year period under investigation, a decreasing trend of erythromycin resistance was observed (28/293 isolates, 9.6 %, in 2012 vs. 16/299, 5.4 %, in 2013; *p* = 0.06).

All resistant GAS were uniformly susceptible to all antibiotics tested except for clindamycin (52.3 %) and tetracycline (50 %). All macrolide-resistant isolates with the M phenotype carried *mef*(A), all three isolates presenting the iMLS_B_ phenotype carried *erm*(A), and all *erm*(B)-positive isolates exhibited the cMLS_B_ phenotype (Table 1). No isolates simultaneously carried more than one resistance determinant. The macrolide resistance cMLS_B_/*erm*(B) phenotype/genotype was the most frequent (52.3 %), followed by M/*mef*(A) (40.9 %) and iMLS_B_/*erm*(A) (6.8 %) (Table 1). The prevalence of macrolide resistance phenotypes/genotypes varied slightly during the study period, with a decrement of M/*mef*(A) from 2012 to 2013, the appearance of iMLS_B_/*erm*(A) during 2013, while cMLS_B_/*erm*(B) remained quite constant and was the most prevalent over time (Table 1).

**Table 1:** Annual and total prevalence of macrolide resistance genes and phenotypes within the 44 macrolide-resistant group A streptococcus (GAS)

Macrolide resistance gene/phenotype	No. of resistant strains (%)		
	2012	2013	2012 + 2013
*mef*(A)/M	14 (50 %)	4 (25 %)	18 (40.9 %)
*erm*(A)/iMLS_B_	0	3 (18.75 %)	3 (6.8 %)
*erm*(B)/cMLS_B_	14 (50 %)	9 (56.25 %)	23 (52.3 %)

### Clonal characterization

Among all GAS isolates collected during the 2-year survey, 25 different *emm* types were identified. *emm* types 12, 89, 1, and 4 were the most prevalent, accounting for about 50 % of all isolates. We found that erythromycin-resistant isolates represented only 13 %, 2.9 %, 4.6 %, and 16.7 % of *emm* type 12, 89, 1, and 4 subgroups, respectively. The clonal characterization of erythromycin-resistant strains is illustrated in Table 2, showing 11 different *emm* types , eight of which represented by two or more erythromycin-resistant strains. The predominant *emm* types were *emm*12 and 4 (11 strains each, 25 %), followed by *emm*11 (seven isolates), which were recovered in both study years and accounted for 66 % of all erythromycin-resistant strains (Table 2). As indicated in Table 2, the relative frequencies of *emm* types encountered during the two study years differed slightly, although not significantly as per Fisher’s exact test analysis performed on the three most prevalent *emm* types. Five *emm* types were found during both years, while six were single year-associated but represented the less frequent *emm* types among erythromycin-resistant isolates (Table 2). In order to assess seasonal variation in macrolide-resistant *emm* types distribution, we divided each year into two seasonal periods (January–March and April–June), but no significant differences were found in the prevalence of each *emm* type (data not shown).

**Table 2:** Cross tabulation of *emm* types, pulsed-field gel electrophoresis (PFGE) clustering, and genotypes/phenotypes of resistance for the 44 macrolide-resistant group A streptococcus (GAS) strains isolated in Italy (2012–2013)

*emm* type	Percentage of susceptible plus resistant isolates within the general population (*n* = 592)	No. of macrolide-resistant isolates per year (%)			PFGE type (no. of isolates)^a^	No. of isolates with macrolide resistance gene			Tetracycline resistance gene (no. of isolates)	Antimicrobial resistance profile (no. of isolates)
		All	2012	2013		*erm*(A)	*erm*(B)	*mef*(A)		
4	11.2	11 (25)	9 (25)	2 (12.5)	1 (9); 2 (2)	0	0	11	none	Ery (11)
12	13.0	11 (25)	7 (32.1)	4 (25)	3 (11)	1	7	3	*tet*(M) (7)	Ery,Cli,Tet (7); Ery (4)
11	1.4	7 (15.9)	4 (14.3)	3 (18.8)	4 (7)	0	7	0	*tet*(M) (7)	Ery,Cli,Tet (7)
1	11.2	3 (6.8)	3 (10.7)	0	5 (3)	0	3	0	none	Ery,Cli (3)
2	1.2	3 (6.8)	2 (7.1)	1 (6.3)	6 (3)	0	0	3	*tet*(O) (3)	Ery,Tet (3)
44	5.1	2 (4.5)	0	2 (12.5)	7 (2)	0	2	0	*tet*(M) (2)	Ery,Cli,Tet (2)
77	0.3	2 (4.5)	0	2 (12.5)	8 (2)	2	0	0	*tet*(O) (2)	Ery,Tet (2)
89	11.8	2 (4.5)	1 (3.6)	1 (6.3)	9 (2)	0	2	0	*tet*(M) (1)	Ery,Cli,Tet (1); Ery,Cli (1)
18	3.6	1 (2.3)	1 (3.6)		10 (1)	0	1	0	none	Ery,Cli (1)
75	2.2	1 (2.3)	1 (3.6)		11 (1)	0	0	1	none	Ery (1)
132	0.2	1 (2.3)	0	1 (6.3)	12 (1)	0	1	0	*tet*(M) (1)^b^	Ery,Cli (1)^b^

^a^All isolates positive to *mef*(A) but the *emm*2 and one of the *emm*12 isolates were genotyped by PFGE using *Cfr9*I restriction enzyme, because their genomic DNA was not digested by *Sma*I

^b^Isolate found to be *tet*(M)-positive but tetracycline-susceptible

Tetracycline-resistant isolates that carried *erm*(B) and *tet*(M) belonged to five different *emm* types, the majority of isolates belonging to *emm* types 12 and 11 (seven isolates each). Three *mef*(A)- and *tet*(O)-positive isolates were *emm* type 2, and the two isolates harboring *erm*(A) and *tet*(O) were *emm* type 77 (Table 2).

PFGE analysis was able to discriminate 12 different types (Table 2). An overall concordance was found between *emm* types and PFGE types (adjusted Wallace coefficient: 0.852; 95 % confidence interval: 0.714–0.991), with each *emm* type being associated with a specific and unique PFGE type, except for the *emm* type 4 group, wherein two different PFGE types were found (Table 2). Also, *emm* type 12, which was the most heterogeneous in terms of macrolide resistance determinants, was associated with a single PFGE type (type 3) (Table 2). It was possible to recognize three major clones, which were represented by more than three isolates and defined by the association of *emm* type, PFGE type, and resistance gene profile (Table 2). Macrolide-sensitive isolates belonging to *emm* types 4, 11, 12, and 89 were randomly chosen and genotyped by PFGE (three isolates for each *emm* type). Their profiles were related to those of the most prevalent macrolide-resistant strains of the corresponding *emm* types, namely PFGE type 1 for *emm* type 4, PFGE type 3 for *emm* type 12, PFGE type 4 for *emm* type 11, and PFGE type 9 for *emm* type 89 (data not shown).

## Discussion

The macrolide resistance rates vary considerably among GAS strains from different countries and over time between <3 % to >26 % [2, 16–20]. In Europe from 2005 onwards, while in some regions macrolide resistance rates continued to remain high with an increasing trend, such as Greece [20], in others, such as Spain, Portugal, France, and Germany, a significant decrease of macrolide resistance rates was reported [14, 21–23].

In Italy, based on regional studies mainly, macrolide resistance rates steadily increased from 9 % in 1992 to 53 % in 1997 [24]. Over the period 2000–2009, the rates continued to remain high in Central Italy, varying between 16 % and 36 % [10, 11]. In those years, Italy was among the regions with the highest levels of erythromycin resistance in Europe. According to the present study, we witnessed, for the first time, a decline in macrolide resistance rates in Central Italy, among GAS isolates over the period 2012–2013, down to 7.4 %.

*erm*(B) was the predominant macrolide resistance gene found, followed by *mef*(A). *erm*(B) was responsible for the increase in macrolide resistance rates observed in Italy during the period 1992–1997 and remained prevalent between 2000 and 2003 [10, 24]. In the present study, the *erm*(A) gene was rarely found, as also reported in Belgium [25].

In this study, among erythromycin-resistant isolates, *emm* types 12, 4, and 11 predominated, accounting for 66 % of all resistant strains. In Italy, while *emm* types 12 and 4 were the most prevalent types among erythromycin-resistant isolates in previous studies, *emm*11 was only rarely found, even among susceptible strains [10, 26]. Associations between *emm* types and macrolide resistance genes resulted to be the same as to those found previously, with only rare exceptions, thus suggesting that, in Italy, few successful clones are associated with macrolide resistance [9, 10, 26]. With only one exception, represented by *emm* type 4 with two different PFGE types, each *emm* type was specifically associated with a distinct PFGE type. The most prevalent *emm* types found among our GAS strains represent also those types frequently detected in different geographical areas [2, 20–22]. An *mef*(A)-positive/*emm*4 clone has been frequently found associated with macrolide resistance in GAS [8, 20, 22], as well as an *erm*(B)-positive/*emm*11 clone that seemed to increase in prevalence in some countries [14, 21, 22, 27]. The finding of specific associations between *emm* type and macrolide resistance genotype and the fact that some *emm* types are never or rarely found in resistant isolates are suggestive of the limited transfer of macrolide resistance determinants within GAS. Nevertheless, rare *emm* type/resistance gene associations have been found in our study. It is the case of the second most prevalent detected clone, the *erm*(B)-positive/*emm*12/PFGE 3, whereas *emm*12 isolates resistant to macrolides have been previously found associated with *mef*(A) [2, 21, 25], and the case of three *emm*1 isolates with *erm*(B), whereas *emm*1-resistant isolates are generally *mef*(A)-positive [22, 27]. The uncommon *emm* types/macrolide resistance associations observed in this study could reflect circulation of different clonal lineages in this geographic area.

The finding of 17 out of 22 (77.3 %) erythromycin- and tetracycline-resistant isolates that carried both *erm*(B) and *tet*(M) could suggest that these isolates may carry conjugative transposons belonging to the Tn916 family, such as Tn3872, Tn6002, Tn6003, Tn1545, and Tn2010, where *erm*(B) and *tet*(M) are genetically associated [13]. These *erm*(B)/*tet*(M) isolates belonged to six different *emm* types, the predominant being *emm* types 12 and 11 (seven isolates each). While the association between *emm* type 11 and *erm*(B)/*tet*(M)-mediated co-resistance has already been reported [27], *emm*12 isolates carrying both *erm*(B) and *tet*(M) are very rare, due to the unusual association of this *emm* type with *erm*(B), as stated above. Three *emm*2 isolates had *mef*(A) and *tet*(O), suggesting that they may carry the transferable chimeric prophage Φm46.1 [28]. The two erythromycin- and tetracycline-resistant isolates of *emm* type 77 harbored *erm*(A) and *tet*(O).

High-level macrolide consumption, especially long-acting macrolides, have been directly associated with an increase of macrolide resistance, due to the antibiotic selective pressure, which could favor the spread of specific macrolide-resistant GAS clones, with changes in macrolide resistance rates and genotypes [19]. In some countries, reduction in macrolide consumption proved to be an important factor responsible for the decline in macrolide resistance rates [22, 23, 25]. In Italy, macrolide consumption remained relatively stable during the last 15 years (1999–2013) [29], although available data refer to the general population and not to the pediatric group only. Also, the consumption of tetracyclines, which have been associated with the increase of macrolide resistance, remained stable [29]. Although we do not have information on antibiotic usage in the patients of this study, the observed decline in macrolide resistance rates seems to not correlate to general macrolide consumption. A recent study from Slovenia indicated even the opposite; that is, a boost in resistance rates among non-invasive GAS, despite a decrease in macrolide use [30]. Thus, besides the presumed association with antibiotic use, other underlying mechanisms influencing the development and spread of antibiotic-resistant GAS isolates are to be considered, such as natural fluctuations in the prevalence of resistant clones and low fitness costs of some erythromycin-resistant clones [25]. We found that the major *emm* types in the overall GAS pharyngeal population circulating in Italy during 2012–2013 were the same as those reported in previous Italian studies, specifically *emm* types 4, 12, and 89, although with differences in their relative frequencies [10, 26]. Some of the most frequent *emm* types were also the most prevalent among erythromycin-resistant strains. PFGE was not able to differentiate resistant and susceptible isolates belonging to the same *emm* type, and this observation seems to indicate that the decrease of macrolide resistance would not be due to a decrement of specific GAS macrolide-resistant clones within a given *emm* type.

This study documents a decline in macrolide resistance rates in Italy, where macrolide resistance has been documented to be high in the past, and it provides useful comparative data for future epidemiological studies across erythromycin-resistant GAS populations.
